# Contrasting dietary patterns remodel gut microbial function and generate multi-omic signatures associated with cardiometabolic markers

**DOI:** 10.1080/19490976.2026.2685381

**Published:** 2026-06-11

**Authors:** Jordan Stanford, Emily C. Hoedt, María Gómez-Martín, Erin D. Clarke, Kerith Duncanson, Tracy Burrows, Clare E. Collins

**Affiliations:** a School of Health Sciences, College of Health Medicine and Wellbeing, The University of Newcastle, Callaghan, NSW, Australia; b Nutrition and Metabolic Health Research Program, Hunter Medical Research Institute, New Lambton Heights, NSW, Australia; c School of Biomedical Sciences & Pharmacy, College of Health, Medicine and Wellbeing, University of Newcastle, Callaghan, NSW, Australia; d Immune Health Research Program, Hunter Medical Research Institute, New Lambton Heights, NSW, Australia; e Centre of Research Excellence in Transforming Gut Health, College of Health, Medicine and Wellbeing, University of Newcastle, Callaghan, NSW, Australia; f School of Medicine and Public Health, The University of Newcastle, Callaghan, NSW, Australia

**Keywords:** Gut microbiome, dietary intervention, randomized crossover feeding trial, microbial functional pathways, multi-omics integration

## Abstract

Diet is a modifiable determinant of gut microbiome composition, yet the impact of contrasting whole-dietary patterns on microbial metabolic capacity and coordinated host metabolic signatures remains incompletely characterized. In a randomized crossover feeding trial, 34 Australian adults were provided with a Healthy Australian Diet (HAD), aligned with national dietary guidelines, and a Typical Australian Diet (TAD), reflecting average population intake for two weeks each, separated by a two-week washout. Fecal microbiome composition and function were assessed using shotgun metagenomics, plasma and urine metabolites by untargeted metabolomics, with cardiometabolic markers including blood pressure, plasma lipids, and glucose quantified. HAD was associated with reduced taxonomic and functional alpha diversity relative to baseline, with no change following TAD. Species-level responses were modest, 105 functional pathways differed between diets, with 99 increasing following HAD, predominantly related to amino acid and nucleotide biosynthesis and vitamin/cofactor metabolism. Multi-omic integration using DIABLO achieved strong discrimination of dietary responses (held-out accuracy 91.7%; permutation *p* = 0.005). In total, 77 individual omic feature-cardiometabolic outcome associations survived FDR correction (*q* < 0.05), spanning microbial gene functions, plasma metabolites, and urinary metabolites linked to cholesterol, blood pressure, and triglyceride responses. These exploratory findings suggest that integrated microbiome-metabolome profiling may capture inter-individual variation in dietary cardiometabolic responses, though replication in larger, independent, robustly designed studies is needed before translational personalized nutrition strategies can be assessed.

## Introduction

Diet is a primary driver of gut microbiome composition and metabolic function, with implications for host cardiometabolic, immune, and neurological health.^1-3^ Plant-rich dietary patterns support beneficial colonic microbiota, including saccharolytic taxa and the production of health-associated metabolites,^3^ ^,^ ^4^ whereas Western dietary patterns, characterized by high intakes of refined carbohydrates and saturated fat, are associated with greater abundance of opportunistic microbes and increased systemic inflammation.^4^ In Australia, poor diet quality accounts for over 7% of the national disease burden.^5^ The latest National Health Survey (2022) indicates worsening diet quality trends, with declining vegetable intake compared with the 2011–2012 survey, and only 6.5% of adults meeting the recommended vegetable intake.^6^ At the same time, discretionary, energy-dense, nutrient-poor foods contribute approximately one-third of daily energy intake.^6^

The gut microbiome is increasingly recognized as a mechanistic intermediary linking diet to host physiology,^3^ in large part through its influence on the circulating metabolome.^3^ Microbial communities metabolize dietary substrates and endogenous components to produce bioactive metabolites, including short-chain fatty acids, bile acid derivatives and tryptophan catabolites, that enter circulation and directly modulate host metabolic, inflammatory and endocrine pathways.^7^ ^,^ ^8^ Plasma and urinary metabolite profiles represent an integrated readout of this host-microbiome metabolic exchange, with alterations in metabolite profiles associated with cardiometabolic risk factors including high plasma cholesterol levels, elevated blood pressure and insulin resistance.^9^ ^,^ ^10^ However, as with other interventions, cardiometabolic responses to dietary modification are not uniform. Individuals consuming the same diet can exhibit markedly different clinical profiles and thus there is a growing recognition of inter-individual variation in gut microbiome and function composition as a contributor to this heterogeneity.^11^ Characterizing these interconnected layers requires multi-omic approaches, in which complementary biological measurements, such as metagenomics, meaning what genes the microbial community encodes; metabolomics meaning what small molecules are present in circulation and urine, and clinical phenotyping are collected from the same individuals and analyzed jointly rather than in isolation.^3^ ^,^ ^11^ By integrating these data types, multi-omic analysis can identify whether changes in microbial function, host metabolites, and clinical outcomes are coordinated,^3^ ^,^ ^11^ and pinpoint which specific features co-vary with one another.

Despite this, much of the existing evidence linking diet, microbiome and metabolome have been dominated by observational studies and free-living interventions constrained by heterogenous dietary adherence or emphasis on isolated foods or nutrients rather than whole dietary patterns, supported by provision of all foods.^1^ ^,^ ^12^ ^,^ ^13^ While the principle that diet influences microbial metabolic function is increasingly recognized, recent reviews have highlighted that controlled feeding trial evidence directly demonstrating this, using measured rather than computationally inferred functional profiles, remains limited,^14-17^ particularly for whole dietary patterns. Emerging evidence suggests that microbial functional responses may be partially decoupled from taxonomic composition, indicating that functional capacity may represent a more sensitive marker of the impact of dietary intake, rather than community structure alone.^2^ ^,^ ^18^ While prior research has predominantly focused on microbial catabolic processes, such as fermentation of dietary substrates,^19^ the extent to which diet influences microbial anabolic capacity, including biosynthesis of essential amino acids, nucleotides, and vitamins,^20^ ^,^ ^21^ remains less well characterized. Whether such diet-induced functional changes are accompanied by coordinated shifts in host circulating metabolites has rarely been examined in cross-over feeding human trials with directly measured microbial function alongside untargeted metabolomics. To address these limitations, we utilized data from a randomized crossover feeding trial comparing a Healthy Australian Diet (HAD), aligned with national dietary guidelines, with a Typical Australian Diet (TAD) reflecting population-level apparent consumption, meaning it is relatively unhealthy. We aimed to determine whether two weeks of consuming these contrasting dietary patterns induces cohort-wide, diet-specific changes in gut microbiome diversity, taxonomic composition, and functional capacity, and whether diet-related shifts in microbial function are coordinated with corresponding changes in plasma and urinary metabolite profiles. A secondary aim was to examine whether the magnitude of individual multi-omic responses to dietary intervention was associated with differential cardiometabolic outcomes, and to identify specific microbial functions and metabolites whose diet-induced changes tracked with changes in clinical markers of cardiovascular risk.

## Methods

### Study design and ethics

This 8-week randomized crossover trial was conducted in healthy Australian adults. Following a two-week run-in period on their habitual diet, participants completed 2 × two-week dietary intervention periods (HAD or TAD) in randomized order, during which all foods and selected beverages were provided. Diet allocation was determined using computer-generated block randomization, stratified by sex and enrollment status (individual or couple). The intervention periods were separated by a two-week washout during which participants resumed their habitual diet. The within-subject design minimized confounding due to inter-individual variability in baseline microbiome composition and enabled robust characterization of integrated host-microbiome metabolic responses to whole dietary patterns.

The study was approved by the Hunter New England (2022/ETH01649) and the University of Newcastle (H-2022-0330) Human Research Ethics Committees. All participants provided written informed consent prior to enrollment. The trial was conducted in accordance with the Declaration of Helsinki and prospectively registered with the Australian New Zealand Clinical Trials Registry (ACTRN12622001321730). The trial protocol and primary outcomes paper have been published.^22^ ^,^ ^23^

### Participants and recruitment

Participants were recruited from the Hunter region (New South Wales, Australia) between September 2022 and May 2023 using multimodal strategies, including study flyers displayed across the University of Newcastle campus, utilizing existing research volunteer registries as well as radio and social media advertisements. Participants were excluded if they were <18 y of age, had food allergies or intolerances, uncontrolled hypertension, type 1 diabetes or insulin-dependent type 2 diabetes, cancer diagnosis requiring active treatment, gastrointestinal disorders, were following a restrictive or therapeutic diet, were pregnant or breastfeeding, consumed excessive alcohol, or were using medications or supplements known to influence metabolic or microbiome outcomes (i.e., antibiotics). As the study was originally designed to identify urinary and plasma metabolites that distinguish a “healthy” dietary pattern (HAD; aligned with current national dietary guidelines) from an “unhealthy” dietary pattern (TAD), the target sample size was at least 20 participants. This estimate was informed by a scoping review of comparable crossover feeding studies.^24^ Fecal microbiome profiling via shotgun metagenomics was pre-specified in the published protocol^23^ as part of the study's multi-omic design, and the integration analyses were outlined in the statistical analysis plan. However, the sample size was determined for the metabolomics aims and therefore the microbiome and multi-omic integration analyses were not independently powered, which should be interpreted as hypothesis-generating.

### Dietary interventions

The two dietary interventions have been described in detail elsewhere.^22^ ^,^ ^23^ In brief, HAD was designed in accordance with the Australian Dietary Guidelines to meet the recommended servings of the five core food groups and Acceptable Macronutrient Distribution Ranges.^25^ In contrast, TAD was modeled on data from the 2020-2021 Apparent Consumption of Foodstuffs report^26^ to reflect apparent population consumption data, characterized by higher intakes of saturated fat, sodium, and free sugars, and lower dietary fiber. All foods and selected snacks were provided to participants during both the HAD and TAD feeding periods to standardize dietary intake. The energy content of food and beverages provided was matched to estimated energy requirements for each individual across the two intervention periods to ensure weight stability. To assess dietary compliance, specific foods containing well-characterized metabolite biomarkers were incorporated into each diet. For example, the HAD was deliberately enriched with wholegrain products, which are associated with metabolites such as gentisate.^27^ ^,^ ^28^ In contrast, dark chocolate (85% cocoa-derived ingredient) was provided daily during the TAD only due to its theobromine content.^29^ ^,^ ^30^ Additionally, participants consumed a daily serving of orange juice during both intervention periods, where its high proline-betaine content served as an objective biomarker of compliance.^31^

### Data collection

Demographic and dietary assessment data were collected online. Baseline demographic information included medical history, current medication and supplement use, smoking status, alcohol consumption, recent changes in diet or physical activity, employment, education, and ethnicity, which were collected via REDCap. At each follow-up time point, participants reported any changes in health status, medication use, or supplement use during the preceding two weeks. Dietary intake during each intervention period was assessed using the Automated Self-Administered 24-hour Dietary Assessment Tool - Australia (ASA24 AUS). Reminders for three 24-hour recalls were sent to participants per intervention period to quantify intake and assess adherence: one during week one and two during week two, aligned with the 24 hours prior to biospecimen collection. For any deviations from the supplied study foods, meals, or beverages, participants were asked to complete food records daily using Easy Diet Diary (Xyris Software, Australia). Habitual dietary intake and diet quality over the previous three months were assessed at baseline using the Australian Eating Survey® (AES®), a validated 135-item semi-quantitative food frequency questionnaire.

Blood pressure measures as well as biospecimen collection (blood, urine, and fecal samples) were collected in person at the Nutrition and Dietetics Clinical Research Laboratory, The University of Newcastle. Blood pressure was measured using the Uscom BP + supra-systolic oscillometric device (Uscom Ltd., Sydney, NSW, Australia), with the cuff positioned on the upper left arm. Participants were seated and rested for 5 minutes prior to measurement, maintaining an upright posture with legs uncrossed and feet flat on the floor. Three readings were obtained at 1-minute intervals. The first reading was discarded, and the mean of the second and third readings was recorded. If either the second or third reading was unavailable, the remaining valid reading was used.

### Biospecimen collection and processing

Participants attended clinical visits following an overnight fast of at least 8 hours and were instructed to abstain from exercise and alcohol for 24 hours prior to their assessment visit. Fasting venous blood samples were collected by a qualified phlebotomist. Clinical biochemistry analyses, including lipid profile and fasting glucose, were performed by NSW Pathology. Additional EDTA-treated blood samples were centrifuged at 3000 rpm for 15 minutes at 4 °C, and plasma, red blood cells, and buffy coat fractions were aliquoted and stored at −80 °C. Participants self-collected midstream urine samples (not first void) in sterile containers. Samples were placed on ice immediately, aliquoted within 2 hours of collection, and stored at −80 °C until analysis. Fecal samples were self-collected by participants within 3 d of clinic visits using at-home collection kits containing Zymo catchment paper and DNA/RNA stabilization tubes. Samples were stored at ambient temperature and returned either at the next clinic visit or via prepaid mail. Upon receipt, samples were stored at −80 °C until processing. Microbial DNA was extracted by researchers from the Hunter Medical Research Institute (HMRI), and sequencing was performed by Microba Life Sciences (Brisbane, Australia).

### Microbiome sequencing and bioinformatics

Shotgun metagenomic sequencing libraries were prepared according to the manufacturer's protocol using the Nextera DNA Flex Library Preparation Kit (20018705) and indexed with IDT for Illumina Nextera DNA Unique Dual Indexes Set A-D (20027213-6). Libraries were pooled in equimolar concentrations and sequenced on an Illumina NovaSeq6000 platform using 2 × 150 bp paired-end chemistry by Microba PTY LTD (Brisbane, Australia). Samples were sequenced to a target depth of 4Gb per sample, with a minimum depth of 3Gb.

Raw sequencing reads were quality filtered, and host DNA was removed using the KneadData pipeline. Taxonomic profiling was performed using MetaPhlAn4 (v4.0.6) using the mpa_vJun23_CHOCOPhlAnSGB_202403 marker database to obtain the most up-to-date species-level resolution, including recently defined species genome bins (SGBs). MetaPhlAn was executed with options enabling estimation of unclassified reads and inclusion of viral taxa, improving representation of overall community composition. For functional profiling, HUMAnN3 (v3.8) was run using precomputed MetaPhlAn taxonomic profiles generated with the mpa_vOct22_CHOCOPhlAnSGB_202212 marker database, which corresponds to the ChocoPhlAn nucleotide database distributed and validated with HUMAnN3. This ensured compatibility between taxonomic pre-screening and nucleotide-level functional mapping, while maintaining reproducibility of functional inference. Gene family abundances were annotated against the UniRef90 database and mapped to metabolic pathways using MetaCyc, with additional regrouping to KEGG orthologs.

### Metabolomic analysis

Plasma and urine metabolomic profiling was conducted by Metabolon Inc. (Morrisville, USA) using ultra-high-performance liquid chromatography-tandem mass spectrometry (UPLC–MS/MS) via their Global Discovery Panel. Plasma and urine samples were analyzed in a single batch, with samples distributed across plates containing internal quality control samples. Urine metabolites were normalized to osmolality. Missing values for both plasma and urine datasets were imputed using the minimum observed value for each metabolite, and all metabolite abundances were scaled to a median of one in accordance with Metabolon's standard processing procedures.^32^

### Statistical analysis

All statistical analyses were conducted in R (v4.4.2). A detailed description of all methods is provided in the extended statistical analysis in Supplementary materials. Species-level abundances, MetaCyc pathway abundances, and KEGG Orthology (KO) gene family abundances were generated as relative abundances. Unclassified or unmapped entries were removed, and relative abundances were renormalized. To reduce sparsity, features were prevalence-filtered prior to downstream analysis: species and KOs were retained if present in ≥20% of samples, and pathways if present in ≥30%. Filtered relative abundance tables were centered log-ratio (CLR) transformed following the addition of a pseudocount to all entries. Plasma and urine metabolomic profiles were log-transformed as provided by Metabolon, Inc. Alpha diversity (observed features, Shannon, Simpson, and Pielou's evenness) was computed from filtered relative abundances using the *vegan* package (v2.6.10). Beta diversity was assessed using Aitchison distance, with participant-stratified PERMANOVA (999 permutations). All univariate analyses used a 2 × 2 crossover linear mixed-effects model (LMM) in the form: outcome ~ treatment × trial + period + sequence + age + sex + (1|participant), fitted with restricted maximum likelihood (REML) and Satterthwaite degrees of freedom using *lme4* (v1.1.36) and *lmerTest* (v3.1.3) packages, following established crossover trial methodology.^33^ Within-treatment changes (post-diet vs. baseline) were estimated from model-derived marginal means. Between-diet effects were assessed using a difference-in-differences (DiD) approach, calculated as the change from baseline to post-TAD minus the corresponding change under the HAD. Estimates and contrasts were obtained using the *emmeans* package (v2.0.0). For per-feature analyses (e.g., individual species, KOs, pathways, or metabolites), *p*-values were corrected for multiple testing using the Benjamini–Hochberg false discovery rate (FDR < 0.05), implemented via the p.*adjust* function in the stats package (v4.4.2). Multi-omic integration was performed using DIABLO as implemented in the *mixOmic*s package (v6.32.0). Three predictor blocks (KOs, plasma metabolites, and urine metabolites) were constructed from within-treatment change scores (Δ = post-diet vs. baseline) and z-score standardized. Discriminant analysis classified samples by dietary treatment (HAD vs. TAD) using subject-blocked 5-fold cross-validation (10 repeats). Feature sparsity (keepX) was optimized via grid search, and classification performance was evaluated against chance by permutation testing (195 permutations). Separately, block sparse partial least squares (block.spls) regression was used via *mixOmic*s package to identify multi-omic features that are associated with markers of cardiometabolic health responses (within-subject DiD; *n* = 32 independent observations), with selected features validated by Spearman correlations and FDR correction.

### Data availability

Host depleted metagenomic data are available and have been deposited under NCBI BioProject accession number PRJNA1470033. NCBI SRA record is accessible with the following link http://www.ncbi.nlm.nih.gov/bioproject/PRJNA1470033. Metabolomic data have been deposited in MetaboLights under accession number MTBLS14664. Additional metadata is available via Zenodo (DOI: 10.5281/zenodo.20196504), with access contingent upon ethics approval, obtaining proposal approval, and completion of a Data Transfer Agreement.

## Results

### Participant characteristics

**Figure 1.**
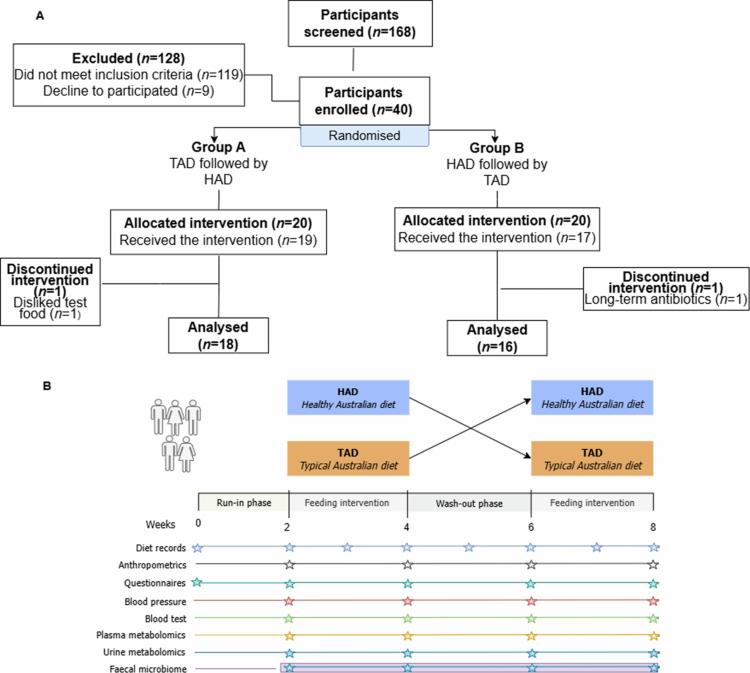
CONSORT flow diagram and overview of data collection procedures.

Of 168 adults screened, 40 were enrolled, and 34 completed the study. Stool samples from two participants (three samples in total) were either not returned or contaminated and therefore excluded from microbiome analyses. Complete paired pre-post biospecimen data for both dietary interventions were available for 32 participants (mean age 38 ± 18 y; 53% female), with 34 contributing at least one paired pre-post dataset (38 ± 18 y; 53% female) and included in the linear mixed model analyses (Figure 1). Baseline participant characteristics (*n* = 34) are summarized in Table 1. Characteristics of participants for all other analytic subsets are provided in Supplementary material Table S1.

**Table 1.** Characteristics of participants at their first study visit (*n* = 34).

Characteristic	Value
**Demographic variables (mean, SD, or *n* (%))**	
Sex, % female (*n*)	18 (52.9%)
Age, years	38.4 ± 18.1
Born in Australia, *n* (%)	23 (67.6%)
**Anthropometric variables (mean, SD)**	
Weight, kg	76.8 ± 15.6
Body Mass Index (BMI), kg/m²	26.5 ± 5.4
**Dietary intake variables (median, IQR)^a^**	
Core foods (%kJ)	71 (63.6–80)
Non-core foods (%kJ)	29 (20–36.4)
Vegetables (servings/d)	3.1 (2.1–4.5)
Grains (servings/d)	2.9 (2.2–3.7)
Fruit (servings/d)	1.4 (0.9–2.1)
Seafood (servings/d)	0.3 (0.1–0.6)
Red meat (servings/d)	0.6 (0.3–0.8)
Dietary fiber (g/d)	28.2 (21.4–33.1)
Energy (kJ/d)	8964 (6769.1–10496.1)
Protein (g/d)	104.3 (65.6–117.5)
Added sugars (g/d)	31.5 (16.2–44.9)
Saturated fat (g/d)	27.5 (20.7–33.2)
Sodium (mg/d)	1840.1 (1534.2–2341.9)

^a^ Dietary variables were derived from Australian Eating Survey® data. Core foods are defined as per the Australian Dietary Guidelines five food groups (grains, vegetables, fruit, dairy, lean meats/alternatives). Non-core foods are defined as energy-dense, nutrient-poor “discretionary” choices, high in saturated fat, added sugars, and sodium. %kJ = percentage energy. SD = standard deviation. IQR = inter-quartile range.

### Contrasting dietary interventions alter gut microbiome diversity and community structure

**Figure 2.**
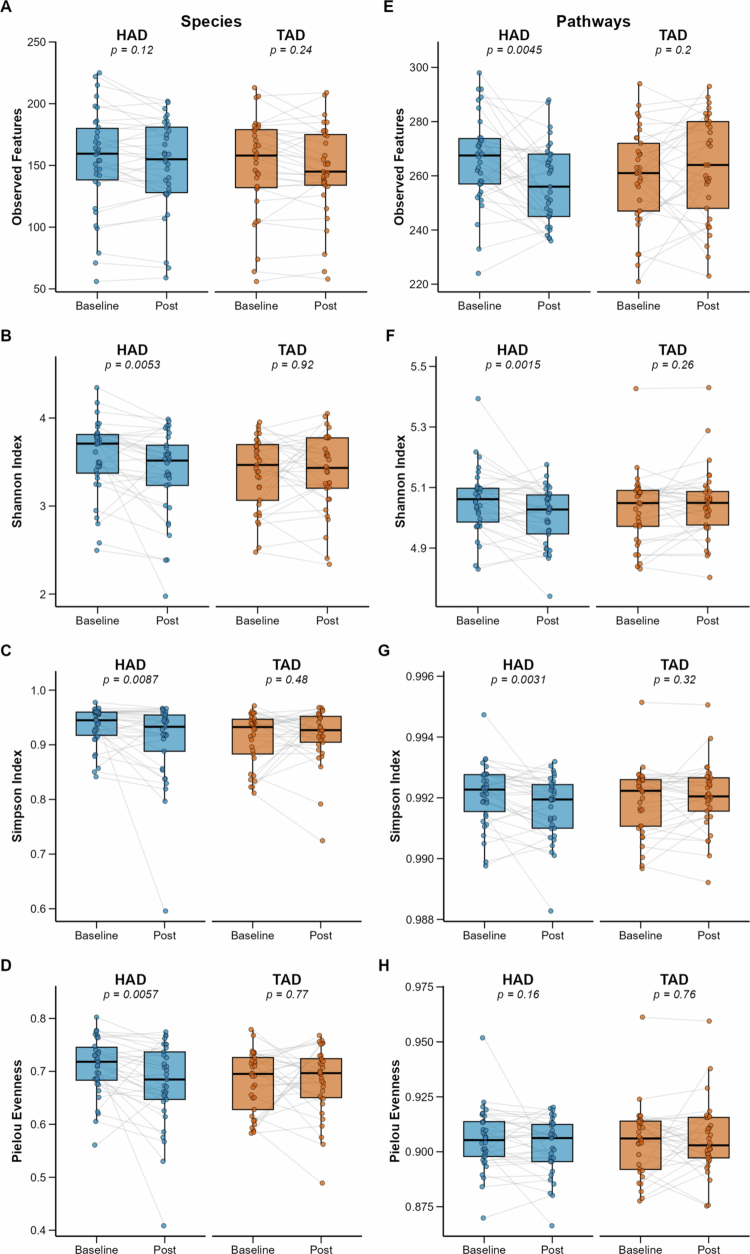
Alpha diversity of microbial species and functional pathways. (A–D) show species-level alpha diversity indices, and (E–H) show microbiome-encoded functional pathway diversity. Boxplots display distributions at baseline and post-intervention within each dietary arm (Healthy Australian Diet [HAD] and Typical Australian Diet [TAD]), with individual participant values overlaid. *p* values were generated using linear mixed-effects models, accounting for crossover design, age, sex, and participant-level random intercepts.

Following HAD, species-level alpha diversity decreased significantly from baseline to post-intervention, as reflected by reductions in Shannon, Simpson, and Pielou evenness indices (Figure 2B–D), indicating reduced community evenness and/or increased dominance of specific taxa. No significant within-diet changes were observed pre-post TAD. Similar patterns were observed at the functional level. Microbiome-encoded pathway diversity (Shannon, Simpson, and observed features) decreased significantly following HAD, with no significant changes after TAD (Figure 2E–G).

Between-diet comparisons confirmed that reductions in alpha diversity were significantly greater following HAD than TAD for Shannon, Simpson, and Pielou evenness indices (*p* = 0.04, 0.019, 0.031, respectively). Likewise, pathway-level declined more under the HAD for observed features, Shannon and Simpson indices (*p* = 0.004, 0.003, and 0.005, respectively). Together, these findings indicate that HAD induced a distinct gut microbiome restructuring, characterized by reduced taxonomic and functional diversity relative to TAD.

**Figure 3.**
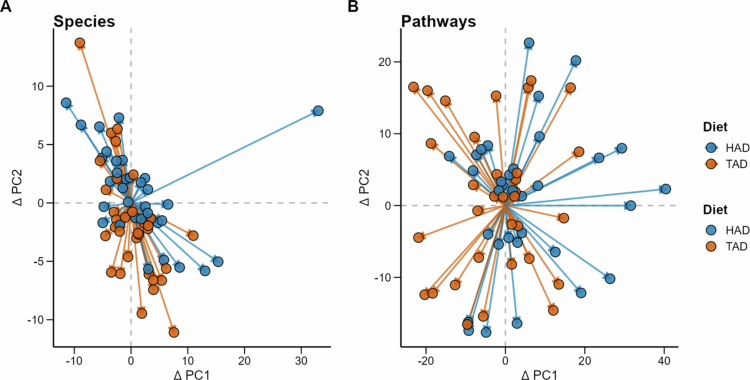
Within-treatment beta-diversity change vectors in a crossover dietary intervention. Arrows represent participant-specific changes from baseline (B) to post-intervention (T) within each dietary arm, visualized in the first two principal coordinates of Aitchison distance based on CLR-transformed species-level (top) and pathway-level (bottom) profiles. Colors indicate treatment (Healthy Australian Diet [HAD] or Typical Australian Diet [TAD]). Vector direction and magnitude reflect the direction and magnitude of compositional change between paired baseline and post-intervention samples from the same individual.

Findings are supported by crossover change-vector plots based on Aitchison distances, illustrating within-individual compositional shifts over time for both microbial species (Figure 3A) and functional pathway composition (Figure 3B). Permutational multivariate analysis of variance (PERMANOVA) revealed a significant diet × time interaction for both microbial species (*R*² = 0.004, *p* = 0.001) and pathway composition (*R*² = 0.009, *p* = 0.004), indicating that overall microbiome structure changed differently, from baseline to post-intervention, in response to both HAD and TAD diets. Although the proportion of variance explained by this interaction was small, such effect sizes are typical in microbiome beta-diversity analyses and reflect consistent, diet-specific restructuring occurring against a background of strong inter-individual variability.

### Species-level taxonomic responses to diet are modest and heterogeneous

**Figure 4.**
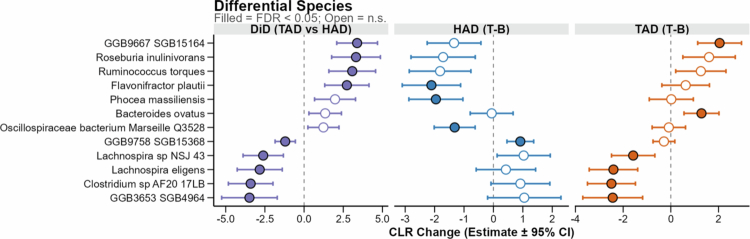
Species-level compositional changes following dietary interventions. Points and horizontal error bars represent estimated changes in center-log-ratio (CLR) relative abundance (±95% confidence intervals) derived from linear mixed-effects models. The left panel shows the between-group difference in change (difference-in-differences; Healthy Australian Diet [HAD] vs. Typical Australian Diet [TAD]), while the middle and right panels display within-group changes from pre- to post-intervention for HAD and TAD arms, respectively. Estimates were obtained using the *emmeans* package and adjusted for crossover design effects, age, sex, and participant-level random intercepts. Filled points indicate species meeting the FDR threshold of <0.05; unfilled points did not meet this threshold.

After prevalence filtering, 298 microbial species were retained for analysis. Of these, 12 species showed significant changes (FDR < 0.05) either within dietary arms (post-diet vs. baseline) or between diets (difference-in-differences; Figure 4). Within the TAD arm, six species changed significantly from baseline. Four species decreased in relative abundance, including *Lachnospira eligens, Clostridium* sp. *AF20 17LB*, while two species increased, including *Bacteroides ovatus.* In contrast, following the HAD intervention, four species showed significant changes relative to baseline. Three species decreased in abundance (*Phocea massiliensis, Flavonifractor plautii, and Oscillospiraceae bacterium Marseille Q3528*), while unclassified Firmicutes species (GGB9758 SGB15368) increased. Between-diet comparisons identified nine species with significantly different baseline-to-post intervention responses, including *Roseburia inulinivorans* and *Ruminococcus torques*, both of which increased to a greater extent under the TAD compared with HAD.

### Microbial functional pathways responded more strongly to dietary intervention than taxonomic composition

**Figure 5.**
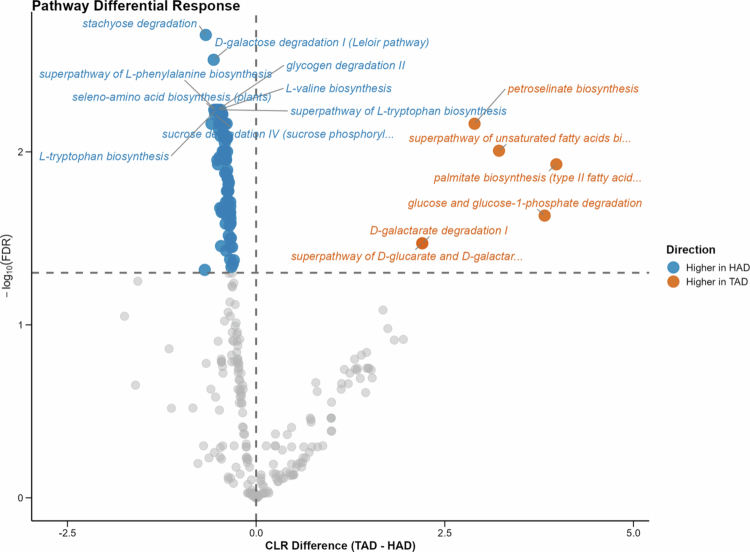
Pathway-level compositional differences in response to dietary interventions. Each point represents a MetaCyc microbial functional pathway. The x-axis shows the between-diet difference in baseline-to-post intervention change (CLR-transformed; Healthy Australian Diet [HAD] vs. Typical Australian Diet [TAD]), and the y-axis displays statistical significance (−log10 FDR-adjusted *p*-value). Positive values indicate a greater change following TAD, whereas negative values indicate a greater change post-HAD. The horizontal dashed line denotes the FDR significance threshold (FDR < 0.05). Pathways meeting this threshold (*n* = 105) are colored: orange indicates greater change post TAD and blue indicates greater change under HAD; non-significant pathways are shown in gray. The 15 most statistically significant between-group differences are annotated (*n* = 9 favoring HAD; *n* = 6 favoring TAD).

After prevalence filtering, 302 microbial functional pathways were retained for analysis. Significant within-diet changes (FDR < 0.05) were observed only in the HAD arm, where 96 functional pathways increased and seven decreased from baseline to post-intervention. Pathways enriched following HAD were primarily related to amino acid biosynthesis, including essential and branched-chain amino acids (e.g., L-valine biosynthesis, *VALSYN-PWY*; L-tryptophan biosynthesis, *TRPSYN-PWY*). Increases were also observed in vitamin and cofactor biosynthesis and salvage (e.g., S-adenosyl-L-methionine salvage, *PWY-6151*; thiamine salvage, *PWY-6897*), central carbon metabolism and selected carbohydrate utilization (e.g., glycolysis, *ANAGLYCOLYSIS-PWY*; galactose metabolism, *PWY-6317*), and nucleotide metabolism (e.g., UMP biosynthesis, *PWY-5686*). In contrast, pathways that decreased post-HAD included fatty acid biosynthesis (e.g., *PWY-5971*, *PWY-5367*, *PWY-6284*), sugar-acid degradation (e.g., D-galactarate degradation, *GALACTARDEG-PWY*), and pyruvate fermentation to propanoate (*PWY-5494*). Between-diet analyses identified 105 pathways with significantly different baseline-to-post change between TAD and HAD (Figure 5). Of these, 99 showed larger increases following HAD, whereas six increased more post-TAD.

Among pathways differing between diets, those showing greater increases post TAD were related to lipid biosynthesis and simple substrate utilization, including palmitate and unsaturated fatty acid biosynthesis (*PWY-5971, PWY-5367*, and *PWY-6284*), sugar-acid degradation (*GALACTARDEG-PWY, GLUCARGALACTSUPER-PWY*), and glucose degradation (*GLUCOSE1PMETAB-PWY*). Overall, TAD was associated with greater enrichment of pathways involved in lipid synthesis and simple substrate utilization relative to HAD.

In contrast, pathways with greater increases following HAD spanned broader functional categories. These included complex carbohydrate utilization and storage (e.g., galactose and stachyose metabolism, *PWY-6317, PWY-6527*; and glycogen metabolism, *PWY-5941*), amino acid biosynthesis (including branched-chain and aromatic amino acid pathways; *VALSYN-PWY, COMPLETE-ARO-PWY*); and nucleotide metabolism (e.g., UMP and purine biosynthesis, *PWY-5686, PWY-7221*). The HAD was also associated with greater enrichment of vitamin and cofactor metabolism (e.g., thiamine salvage, *PWY-6897;* and folate-related pathways) as well as pathways involved in cell wall and structural component biosynthesis, such as peptidoglycan and glycoconjugate synthesis.

### Integrated multi-omic signatures discriminate between dietary patterns

To determine whether gut microbial function and the host metabolome are jointly restructured by these dietary patterns, we applied DIABLO to integrate three omic blocks (microbial-encoded genes (KOs), plasma metabolites, and urine metabolites) using within-subject change scores (post-diet vs. baseline) from 32 participants with complete multi-omic data. The model achieved strong discrimination between HAD and TAD dietary responses (held-out balanced error rate = 0.08, accuracy = 91.7%), supported by permutation testing (*p* = 0.005, 195 permutations; null BER = 0.49, Supplementary Materials).

**Figure 6.**
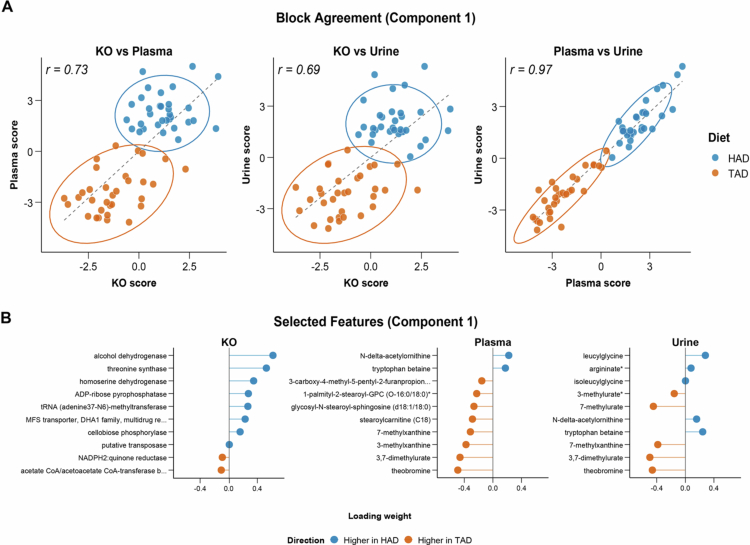
Multi-omic integration using DIABLO identifies diet-specific features across microbial functions and host metabolites. (A) Pairwise scatterplots of block-specific latent scores for Component 1, showing agreement between microbial functional genes (KO), plasma metabolites, and urinary metabolites. Each point represents one participant's within-subject change score (post-diet vs baseline), colored by dietary assignment (Healthy Australian Diet [HAD] = blue; Typical Australian Diet [TAD] = orange). *r* = Pearson correlations between block scores are shown; Ellipses represent 95% confidence regions. (B) Loading weights for features selected on Component 1 within each omic block. Positive loadings (blue) indicate features with greater increases following HAD; negative loadings (orange) indicate features with greater increases following the TAD.

Sample plots confirmed clear separation of HAD and TAD responses along Component 1 across all three omic blocks (Figure 6A). Pairwise correlations between block-specific latent scores were high (plasma–urine metabolites *r* = 0.97; KO-plasma *r* = 0.73; and KO-urine *r* = 0.69), indicating strong concordance of the dietary signal across -omic layers. The near-perfect plasma–urine metabolite correlation suggests overlapping metabolomic responses, while the strong but lower KO-metabolite correlations are consistent with microbial function contributing to, but not fully explaining, the metabolomic shift.

Component 1 captured dominant dietary contrasts (Figure 6B, Figure S1 Supplementary materials). Among microbial functions, alcohol dehydrogenase (K18369; loading = 0.61) and threonine synthase (K01733; loading = 0.52) showed the strongest positive loadings, indicating greater increases from baseline following HAD, alongside homoserine dehydrogenase (K00003; 0.34), ADP-ribose pyrophosphatase (K01515; 0.27), and an MFS multidrug transporter (K08153; 0.22). Acetate CoA-transferase (K01035; −0.11) and NADPH_2_:quinone reductase (K00344; −0.10) loaded in the opposite direction. In both plasma and urine, xanthine metabolites (downstream cocoa/caffeine-related metabolites) such as theobromine, 3,7-dimethylurate, 7-methylxanthine, and 3-methylxanthine, dominated Component 1 with strong negative loadings (–0.31 to –0.50), aligning with higher increases from baseline post-TAD. Loading in the opposite direction, such as tryptophan betaine (plasma 0.17; urine 0.24) and N-delta-acetylornithine (plasma 0.22; urine 0.16) appeared in both biofluids, indicating coordinated shifts in tryptophan and arginine-ornithine metabolism. Several lipids, including stearoylcarnitine (C18), glycosyl-N-stearoyl-sphingosine, and a plasmalogen (1-palmityl-2-stearoyl-GPC), also contributed to the plasma signature.

### Multi-omic signatures were associated with individual cardiometabolic response to dietary intervention

To determine whether the magnitude of diet-induced multi-omic shifts was associated with inter-individual variation in cardiometabolic response, we employed block sparse partial least squares regression using within-subject dietary differences (ΔTAD − ΔHAD; *n* = 32) across microbial gene functions, plasma metabolites, and urine metabolites to model associations with nine clinical markers. Each data point represents one participant's paired difference between dietary periods, such that positive values indicate higher levels following TAD and negative values indicate higher levels following HAD.

**Figure 7.**
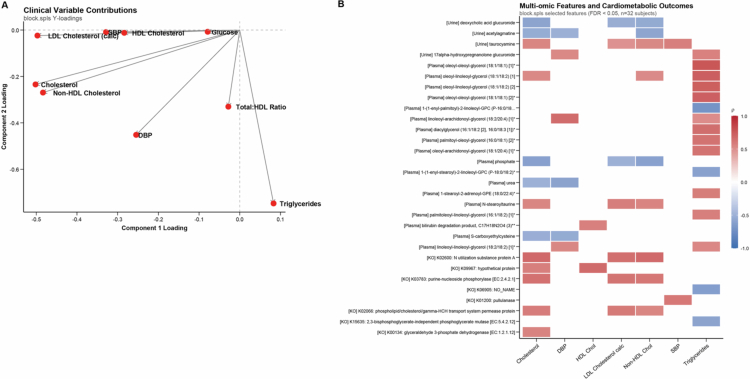
Exploratory associations between multi-omic features and cardiometabolic responses. Block sparse partial least squares (block.spls) regression was used to identify multi-omic features whose within-subject changes (ΔTypical Australian Diet [TAD] − ΔHealthy Australian Diet [HAD]; *n* = 32 subjects) covaried with differential cardiometabolic responses. (A) Clinical variable contributions (Y-loadings) from the block.spls model. Arrow direction and length indicate the strength and direction of each cardiometabolic marker's contribution to the first two latent components. Component 1 captured variance predominantly in cholesterol markers (total, LDL, non-HDL) and systolic blood pressure, while Component 2 was driven by triglycerides, diastolic blood pressure, and total:HDL ratio. (B) Heatmap of Spearman correlations between individual block.spls-selected features and clinical outcomes. Features are grouped by omic block: microbial-encoded genes (KO, bottom), plasma metabolites (middle), and urinary metabolites (top). Red cells indicate positive associations, meaning that participants who showed larger increases in a given feature between diets also tended to show larger increases in the corresponding cardiometabolic marker. Blue cells indicate negative associations. Only features with at least one association surviving FDR correction (q < 0.05) are shown.

The first latent component captured shared variance predominantly in cholesterol, LDL, and blood pressure markers, as indicated by the Y-loadings structure (Figure 7A), while an independent second component loaded most strongly on triglycerides and DBP. Leave-one-out cross-validation of composite scores showed directionally consistent but non-significant associations for Component 1 (e.g., LDL cholesterol *ρ* = −0.29, *p* = 0.11; DBP *ρ* = −0.28, *p* = 0.12; *n* = 32). However, the Component 2 composite score was significantly associated with total cholesterol (*ρ* = -0.41, *p* = 0.02) and non-HDL cholesterol (*ρ* = −0.44, *p* = 0.01) out of sample, with a borderline association with triglycerides (*ρ* = −0.33, *p* = 0.06), suggesting this axis captures shared variance in lipid responses (Figure S2, Supplementary materials). In total, 77 individual feature-cardiometabolic outcome associations across all three omic blocks survived FDR correction (*q* < 0.05; Figure 7B).

Among microbial-encoded genes, participants whose N utilization substance protein A (K02600) increased more between diets also showed larger between-diet increases in total (*ρ* = 0.68, FDR = 0.003), non-HDL (*ρ* = 0.67, FDR = 0.003), and LDL (*ρ* = 0.65, FDR = 0.004), making it the most consistently cholesterol-associated microbial feature. Purine-nucleoside phosphorylase (K03783) and phospholipid/cholesterol transport permease (K02066) showed similar patterns, where greater between-diet shifts in these genes tracked with greater shifts in cholesterol markers (K03783 *ρ* = 0.61–0.64, FDR < 0.01; K02066 *ρ* = 0.59–0.60, FDR < 0.014). In contrast, an unnamed gene (K09967) showed the strongest association with HDL cholesterol (*ρ* = 0.67, FDR = 0.003), where participants with greater between-diet increases in this gene also showed greater increases in HDL. Pullulanase (K01200), a starch-degrading enzyme, was associated with SBP independently of lipid markers (*ρ* = 0.61, FDR = 0.009), with greater between-diet shifts in this gene tracking with larger between-diet differences in blood pressure.

In the urinary metabolome, participants whose deoxycholic acid glucuronide increased more between diets tended to have lower between-diet differences in total- (*ρ* = –0.58, FDR = 0.014), non-HDL (*ρ* = -0.55, FDR = 0.024), and LDL cholesterol (*ρ* = −0.53, FDR = 0.031), making it the most consistently lipid-associated urinary metabolite. Acetylagmatine showed a similar inverse pattern. Participants whose urinary acetylagmatine increased more showed smaller between-diet increases in non-HDL (*ρ* = −0.58, FDR = 0.015) and total cholesterol (*ρ* = −0.54, FDR = 0.028), as well as DBP (*ρ* = −0.51, FDR = 0.041). In the opposite direction, participants whose urinary taurocyamine increased more between diets also showed larger increases in SBP (*ρ* = 0.57, FDR = 0.015), total cholesterol (*ρ* = 0.55, FDR = 0.025), non-HDL cholesterol (*ρ* = 0.53, FDR = 0.030), and LDL cholesterol (*ρ* = 0.49, FDR = 0.048). Urinary cystine showed a similar positive pattern with LDL cholesterol (*ρ* = 0.52, FDR = 0.031), total cholesterol (*ρ* = 0.52, FDR = 0.035), and DBP (*ρ* = 0.50, FDR = 0.043).

In plasma, participants whose phosphate levels increased more between diets tended to have lower between-diet differences in total (*ρ* = −0.61, FDR = 0.009), non-HDL (*ρ* = −0.60, FDR = 0.010), and LDL cholesterol (*ρ* = −0.51, FDR = 0.041). In contrast, those whose N-stearoyltaurine increased more also showed larger increases in LDL cholesterol (*ρ* = 0.58, FDR = 0.015), non-HDL cholesterol (*ρ* = 0.55, FDR = 0.023), and total cholesterol (*ρ* = 0.54, FDR = 0.025). Plasma urea was inversely associated with DBP (*ρ* = −0.59, FDR = 0.013) and total cholesterol (*ρ* = −0.50, FDR = 0.043), where participants with greater between-diet increases in urea tended to show more favorable profiles for these markers. Multiple diacylglycerol species, including oleoyl-oleoyl-glycerol and oleoyl-linoleoyl-glycerol isomers, were the dominant features on the second component, with participants showing greater between-diet increases in these lipid species, with greater increases in triglycerides also (*ρ* = 0.72–0.76, FDR < 0.001).

## Discussion

This randomized crossover feeding trial provides evidence that short-term consumption of nutritionally opposing dietary patterns is associated with shifts in gut microbial metabolic function that are more consistent than changes in taxonomic composition. Using integrated multi-omic profiling, the results indicated that following a two-week dietary intervention was associated with diet-specific differences in microbial functional genes and plasma and urinary metabolites. The magnitude of these multi-omic differences varied between individuals, with specific microbial genes and metabolites associated with between-diet differences in plasma cholesterol, triglyceride, and blood pressure responses.

The observed reductions in both taxonomic and functional alpha diversity following HAD add to the growing evidence questioning the simplistic assumption that higher microbial diversity invariably indicates a healthier gut ecosystem.^34^ ^,^ ^35^ Reduced diversity is frequently associated with disease states, including inflammatory bowel disease, obesity, and type 2 diabetes.^35^ However, these associations are predominantly from observational comparisons where reduced diversity co-occurs with inflammation, barrier dysfunction, and loss of key functional pathways.^36^ ^,^ ^37^ Importantly, short-term high fiber and healthy diet interventions in healthy adults have also led to reduce alpha diversity despite conferring metabolic benefit.^38^ Further, a meta-analysis of higher fiber randomized control trials (RCTs) found no consistent effect on alpha diversity, even when functional markers such as butyrate production increased.^39^ In the present trial, diversity reductions were driven by changes in Shannon and Simpson indices reflecting community *evenness* rather than species richness alone,^34^ ^,^ ^36^ and occurred alongside enrichment of biosynthetic functional capacity. This decoupling of evenness from functional capacity is consistent with functional redundancy in microbial communities, whereby substantial shifts in community structure can occur with preserved or even enriched metabolic pathway capacity.^40^ ^,^ ^41^ Rather than indicating dysbiosis, this pattern is consistent with functional reconfiguration, whereby dietary patterns providing abundant, complex substrates may favor a subset of metabolically adaptive taxa, reducing community evenness, without evidence of widespread species loss or loss of key functional pathways characteristic of disease-associated diversity reductions.^42-45^ However, we note that this pattern is also consistent with short-term substrate-driven adaption, and that for some participants, the likely similarity of their habitual background diets to TAD may have created an inherent asymmetry whereby TAD represents dietary continuity, while HAD represents genuine “healthy” perturbation.

Species-level taxonomic responses were modest, with only nine species showing differential abundance between diets, highlighting the limited sensitivity of species-level composition to short-term dietary modification and perhaps the resilience of an established microbial community adapting to available substrates. *Roseburia* spp., such as *R. inulinivorans*, a butyrate-producing species, showed greater increases following TAD. This species is known to be enriched by certain fiber types and acts as a primary degrader for specific glycans, but its response differs by fiber type and context,^46^ ^,^ ^47^ which may not have been selectively enriched in the HAD. *F. plautii,* capable of short-chain fatty acid production and flavonoid degradation, was similarly higher following TAD. This may reflect adaptation to increased cocoa product consumption, as dark chocolate was provided for daily consumption as an indicator food in this particular dietary pattern. Though this species has also been linked to depletion of protective flavonoids in colorectal cancer,^48^ illustrating the context-dependent interpretation of species-level changes. *Ruminococcus torques*, a mucin-degrading species previously linked to gut barrier thinning and inflammatory bowel disease,^49^ ^,^ ^50^ showed greater increases following TAD. This may indicate a microbial profile more reliant on host-derived substrates under this dietary pattern characterized by high simple sugars and reduced fiber intake. While other controlled feeding interventions have shown more significant gut microbiota changes within just days,^51^ ^,^ ^52^ these studies compared entirely plant-based versus animal-based diets, whereas both interventions in the present trial were omnivorous, which may explain the more modest composition shifts. In this context, two weeks of dietary change may have been insufficient to drive substantial species loss or replacement, instead preferentially altering the function of the existing community.

This interpretation is supported by PERMANOVA results, which showed stronger diet-associated effects on functional pathway composition relative to taxonomic composition, suggesting that microbial function is likely more responsive to dietary modulation than community membership over shorter timeframes. Of the 99 pathways showing greater increases following HAD relative to TAD, most were biosynthetic. This included essential and branched-chain amino acid biosynthesis (L-valine, L-isoleucine), aromatic amino acid biosynthesis (particularly tryptophan), nucleotide biosynthesis (UMP and de novo purine pathways), and vitamin and cofactor metabolism (thiamine salvage, S-adenosyl-L-methionine, and folate-related pathways). These biosynthetic changes were accompanied by increased complex carbohydrate utilization (stachyose and galactose degradation, glycolysis variants, glycogen degradation), potentially consistent with microbiome retooling its metabolic machinery to match a substrate environment rich in diverse fiber types, plant proteins and complex carbohydrates.^53-55^ Enrichment of cell wall and polysaccharide biosynthesis pathways (peptidoglycan maturation, dTDP-L-rhamnose, colanic acid) also suggests increased microbial growth and structural investment.^53^ Taken together, these patterns are consistent with enhanced microbial biomass production rather than metabolic stress. In contrast, TAD was characterized by relatively greater increases in lipogenic pathways, including fatty acid and palmitate biosynthesis, as well as sugar-acid degradation and glucose degradation. This pattern suggests a profile oriented toward simpler substrate utilization and lipid-related processes,^53^ ^,^ ^56^ consistent with the higher total fat and added sugar content characteristic of the TAD. While microbiome research has traditionally focused on catabolic activities such as fiber fermentation, findings from this trial draw attention to a less well-characterized dimension of microbial function,^57^ ^,^ ^58^ which is the capacity for diet-responsive biosynthesis of amino acids, nucleotides, and vitamins.

The coordinated nature of these functional changes raised the question of whether they are accompanied by corresponding shifts in the host metabolome. Integration of microbial-encoded functional genes with plasma and urine metabolites using DIABLO identified multi-omic features that jointly discriminated dietary patterns, suggesting that diet-associated changes extend across multiple biological layers rather than being confined to individual data types. The most prominent feature of the first discriminatory component was a cluster of xanthine metabolites (theobromine, 3,7-dimethylurate, and methylxanthines) detected in both plasma and urine. These metabolites are downstream products of caffeine metabolism, and theobromine is abundant in cocoa.^59^ ^,^ ^60^ As dark chocolate was provided daily in TAD, this signal likely reflects differential dietary exposure rather than a purely microbiome-driven effect. However, microbial alcohol dehydrogenase (K18369) and threonine synthase (K01733) were loaded on the same component, suggesting that microbial metabolic activity co-varied with the xanthine signal. Emerging evidence implies that gut microbes participate in purine and xanthine metabolism,^61^ ^,^ ^62^ raising the possibility that both dietary exposure and microbial activity are plausible contributors. The first component also highlighted coordinated shifts in amino acid and lipid metabolism. Tryptophan betaine (hypaphorine), a plant-derived metabolite found in legumes and pulses, appeared in both biofluids and likely reflects greater plant intake under HAD.^63^ N-delta-acetylornithine co-occurred with microbial threonine and homoserine biosynthesis enzymes (K01733, K00003), suggesting coordinated shifts in host and microbial nitrogen metabolism.^63^ ^,^ ^64^ A second independent component was characterized by acyl-glutamine and acyl-glycine conjugates (e.g., hexanoylglutamine, 4-methylhexanoylglutamine, 3-hydroxybutyroylglycine), metabolites generated during mitochondrial fatty acid oxidation.^65^ ^,^ ^66^ Their co-occurrence with microbial carbohydrate transport genes raises the possibility that diet-induced changes in microbial substrate utilization influence host lipid metabolism, although this hypothesis requires further validation. These two components may capture different layers of the dietary response, the first reflecting direct dietary exposure and substrate-driven microbial adaptation, and the second a downstream metabolic consequence, potentially reflecting how specific diet-microbiome interactions operate through both direct and indirect metabolic routes.

In this study, specific microbial genes and metabolites were associated with how much each participant's cardiometabolic markers changed between diets. Among the 77 associations that survived FDR correction, three patterns emerged, each linked to a different aspect of cardiometabolic risk with largely, though not completely, distinct features. The first pattern involved features associated primarily with cholesterol markers. Urinary deoxycholic acid glucuronide, a bile acid produced by gut microbiota and subsequently conjugated by the host liver, showed inverse associations with total, non-HDL, and LDL cholesterol. This direction is compatible with known roles of secondary bile acids in modulating cholesterol homeostasis via FXR and TGR5 signaling.^67^ ^,^ ^68^ In contrast, microbial-encoded genes phospholipid/cholesterol transport permease (K02066) and purine-nucleoside phosphorylase (K03783) were positively associated with the same lipid markers, suggesting distinct components within microbial bile acid and cholesterol handling may influence host cholesterol in opposing directions.^69^ ^,^ ^70^ However, NusA (K02600), a microbial gene involved in gene expression rather than bile acid metabolism, was the strongest single microbial feature linked to cholesterol, implying that pathways beyond bile acid signaling may also contribute to inter-individual cholesterol variation. The second pattern involved a smaller set of features that bridged both cholesterol and blood pressure outcomes. Urinary taurocyamine was associated with SBP and with total, non-HDL, and LDL cholesterol, while acetylagmatine was inversely associated with non-HDL cholesterol, total cholesterol, and DBP, and cystine showed a similar cross-domain pattern. These cross-domain features are consistent with taurine release during microbial bile salt hydrolase (BSH) -mediated deconjugation and with evidence that BSH-active microbes and bile acid signaling influence both cholesterol and vascular physiology.^67^ ^,^ ^68^ The third pattern was dominated by plasma diacylglycerol species, which showed the strongest effect sizes in the dataset, predominantly with triglycerides, and are known activators of protein kinase C isoforms involved in hepatic lipogenesis and insulin resistance.^71^ ^,^ ^72^ Plasmalogens were inversely associated with triglycerides, suggesting opposing lipid remodeling.^9^ ^,^ ^73^ These largely non-overlapping patterns suggest that the dietary influence on cardiometabolic physiology via the microbiome may operate through multiple, partially distinct routes^74^ rather than a single generalized metabolic shift.^9^ While not all associations clustered within these themes, the convergence of specific microbial genes and metabolites on distinct, biologically plausible clinical outcomes generates testable hypotheses for mechanistic follow-up. These include whether microbial BSH activity mediates the observed associations with cholesterol and blood pressure, and whether the diacylglycerol-plasmalogen balance reflects diet-driven lipid remodeling.

A key strength of this study is the crossover feeding design with all meals provided, which controls for inter-individual confounding and dietary adherence variability. However, several limitations should be considered when interpreting these results. The sample size (*n* = 32–34, depending on the analysis) is limited relative to the dimensionality of the multi-omic data, and the generalizability of these model-derived findings should be interpreted cautiously. In particular, it restricts the ability to conduct adequately powered subgroup analyses, including sex-stratified analyses. Although sex and age were included as covariates, larger studies are required to robustly examine these stratified effects. We note, however, that the crossover design provides within-subject control that increases statistical efficiency relative to a parallel-arm design of equivalent size, and that the core feature-level evidence, 77 FDR-corrected Spearman correlations computed on within-subject difference-of-differences, comprises simple bivariate tests on independent observations (*n* = 32), not model-dependent composite scores. Leave-one-out cross-validation of block.spls composite scores demonstrated that Component 1 composites showed directionally consistent but non-significant out-of-sample associations, while Component 2 retained significance for total and non-HDL cholesterol. All multi-omic integration findings should therefore be considered hypothesis-generating and require replication in larger, independent cohorts. The DIABLO analysis was designed to prioritize covariance between microbial genes and metabolites over covariance between plasma and urine metabolites, reflecting the primary interest in whether microbial functions track with metabolomic changes. This choice may have led to under-selection of microbial features that contribute to dietary discrimination through pathways not reflected in the measured metabolome. The strong dietary classification (held-out accuracy 91.7%), while supported by permutation testing confirming the result exceeded chance expectation, should be interpreted in the context of the small sample and the relatively few features retained after sparse selection. The block.spls analysis identifies associations and not causal relationships. While crossover design controls for stable between-subject differences, it cannot establish whether microbial changes drive metabolomic changes, or vice versa, or whether both respond independently to the same dietary stimulus. Finally, the short intervention duration does not allow assessment of whether these changes persist beyond two weeks, and the controlled feeding design, while maximizing internal validity, may not reflect real-world dietary adherence.

## Conclusion

In this crossover feeding trial, two weeks of contrasting dietary patterns were associated with changes in gut microbial metabolic function, with functional pathways responding more consistently than species-level taxonomy. The HAD was associated with reduced microbial diversity alongside enrichment of biosynthetic pathways, including amino acid, nucleotide, and vitamin metabolism, a pattern consistent with functional reconfiguration in response to dietary substrate availability. Multi-omic integration identified diet-discriminating features spanning microbial genes, plasma, and urinary metabolites with 77 microbial genes and metabolites significantly associated with between-diet differences in plasma cholesterol, triglycerides, and blood pressure after FDR correction, converging on bile acid, taurine, and diacylglycerol pathways. These exploratory findings suggest that integrated microbiome-metabolome profiling may capture inter-individual variation in dietary cardiometabolic responses. However, the sample size limits generalizability and warrants replication in larger, independent, robustly designed studies before the biological or translational significance of these associations can be established.

## Supplementary Material

Supplementary Material SUPPLEMENTARY_MATERIALS.docx

## Trial registration

The trial was prospectively registered with the Australian New Zealand Clinical Trials Registry at https://www.anzctr.org.au (ACTRN12622001321730).
