# Supplementary material for: Contrasting dietary patterns remodel gut microbial function and generate multi-omic signatures associated with cardiometabolic markers
Source: Gut Microbes. 2026 Jun 11;18(1):2685381. doi: 10.1080/19490976.2026.2685381 (PMC13274150; doi:10.1080/19490976.2026.2685381)
Supplement: Supplementary Material — SUPPLEMENTARY_MATERIALS.docx [file KGMI_A_2685381_SM6089.docx]

**SUPPLEMENTARY MATERIALS**

**Contrasting dietary patterns remodel gut microbial function and generate multi-omic signatures linked to cardiometabolic markers**

**Table of contents**

| **Section / Title** | **Item** | **Page(s)** |
| --- | --- | --- |
| Extended statistical analysis methods | - | 2-6 |
| Participant characteristics for analytical subsets at baseline (first visit) | Table S1 | 7 |
| DIABLO model classification performance and permutation testing results | Table S2 | 8 |
| DIABLO Circos plot (Component 1) | Figure S1 | 9 |
| Multi-omic associations with cardiometabolic outcomes (Component 2) | Figure S2 | 10 |

**Item S1. Extended statistical analysis methods:**

**Study design and data overview**

This study was a 2×2 crossover design in which each participant received both the Healthy Australian Diet (HAD) and the Traditional Australian Diet (TAD) in randomised sequence, with measurements at baseline and post-intervention for each treatment period. All analyses were performed in R (v4.4.2). The crossover design was validated by confirming that each participant contributed observations across all four condition cells (HAD-Baseline, HAD-Post, TAD-Baseline, TAD-Post); participants lacking complete data were flagged and handled appropriately in each analysis.

**Multi-omic data layers**

Five data blocks were analysed: (i) gut microbiome taxonomic profiles at species resolution, (ii) metagenomic functional pathways (MetaCyc), (iii) KEGG Orthology (KO) gene families, (iv) plasma metabolomics, and (v) urine metabolomics. Additionally, clinical cardiometabolic variables (systolic and diastolic blood pressure, total cholesterol, LDL cholesterol, HDL cholesterol, non-HDL cholesterol, total:HDL ratio, triglycerides, and fasting glucose) were extracted from participant metadata.

**Data pre-processing**

**Metagenomic profiling**

Shotgun metagenomic sequences were taxonomically profiled using MetaPhlAn4 (v4.0.6) yielding relative abundance estimates at all taxonomic ranks. Functional profiling was performed using HUMAnN3 (v3.8), producing unstratified pathway abundance tables (MetaCyc) and KEGG Orthology (KO) gene family abundances.

**Microbiome data cleaning and transformation**

**Quality filtering:** Non-biological bookkeeping rows were removed from each feature table: UNCLASSIFIED entries from taxonomic profiles, and UNMAPPED, UNINTEGRATED, and UNGROUPED entries from pathway and KO tables. After removal, each sample was renormalised to a relative abundance sum of 1.0 to restore compositional validity.

**Prevalence filtering:** To remove rare features unlikely to yield stable statistical estimates, tiered prevalence thresholds were applied: features present in fewer than 20% of samples were removed for species, and KEGG KO tables, and fewer than 30% for pathway tables.

**Centred log-ratio transformation:** Filtered relative abundance tables were transformed using the centred log-ratio (CLR), which maps compositional data from the simplex to unconstrained real space, enabling the use of standard multivariate statistics. Prior to transformation, a pseudo-count of half the table-wide minimum non-zero value was added to all entries to handle structural zeros. The CLR for each sample was computed as the log of each value minus the mean of all log-values in that sample. The resulting CLR matrices (samples × features) were used for all downstream analyses including linear mixed models, PERMANOVA, DIABLO, and block.spls.

**Metabolomics data**

Plasma and urine metabolomic profiles were provided as log-transformed peak intensities from Metabolon, Inc. Missing values, assumed to represent metabolites below the limit of detection, were imputed with the per-feature observed minimum, a conservative approach standard in untargeted metabolomics. Zero-variance features were removed. Metabolite identifiers (CHEM_ID) were mapped to chemical names and super-pathway/sub-pathway annotations using Metabolon annotation files. This enabled biological interpretation of downstream results at the chemical and pathway level.

**Alpha diversity**

Alpha diversity was quantified from abundance profiles using four metrics: observed features (richness), Shannon index, Simpson index, and Pielou’s evenness. These were computed externally using the vegan R package.

**Linear mixed-effects models**

All univariate analyses (alpha diversity metrics, per-feature differential abundance, per-feature differential metabolite levels) were conducted using linear mixed-effects models (LMMs) fitted via restricted maximum likelihood (REML) using the lme4 and lmerTest R packages. The model specification followed standard 2×2 crossover trial methodology:

*Y ~ treatment × trial + period + sequence + age + sex + (1 | participant_id)*

Where *treatment* (HAD/TAD) and *trial* (Baseline/Post) were fully crossed (the treatment×trial interaction represents the difference-in-differences, DiD); *period* and *sequence* were included as additive nuisance terms to account for crossover carry-over and period effects; *age* and *sex* were fixed covariates; and *participant_id* was a random intercept accounting for within-subject correlation across repeated measures. Type III F-tests with Satterthwaite denominator degrees of freedom were obtained via lmerTest. Model convergence and singularity were checked for every model.

Estimated marginal means (EMMs) were computed using the emmeans package. Two families of contrasts were extracted: (i) within-treatment changes (post diet - baseline for each diet), and (ii) the between-treatment difference-in-differences (DiD), representing the differential change response to TAD versus HAD.

**Alpha diversity analysis**

LMMs as specified above were fitted to each alpha diversity metric (observed features, Shannon, Simpson, Pielou’s evenness) at three feature levels: species, pathways, and KOs. P-values for within-treatment contrasts and DiD contrasts were reported without multiple comparison correction, as these represent a small number of pre-specified, mathematically correlated diversity descriptors (Shannon, Simpson, and Pielou’s evenness are algebraically related through the underlying abundance distribution) rather than a discovery screen across independent hypotheses.

**Beta diversity analysis**

Beta diversity was assessed using Aitchison distance, which is the Euclidean distance in CLR-transformed space and is the appropriate distance metric for compositional data. Principal coordinates analysis (PCoA) was performed on Aitchison distance matrices for ordination.

Permutational multivariate analysis of variance (PERMANOVA) was conducted using the *adonis2* function in vegan (Oksanen et al., 2024) with the full crossover model terms (treatment × trial + period + sequence + age + sex), 999 permutations, and permutations stratified by participant to respect the repeated-measures design. Marginal sums of squares were used to assess each term independently.

Multivariate homogeneity of dispersions (PERMDISP) was tested using *betadisper* and *permutest* (999 permutations) to verify that significant PERMANOVA results were not confounded by differences in within-group dispersion.

**Per-feature differential analysis**

Feature-level differential analysis was performed for species (CLR-transformed abundances) and functional pathways (CLR-transformed). Each feature was independently modelled using the LMM described above. Within-treatment (post diet - baseline) and DiD contrasts were extracted via emmeans. P-values were corrected for multiple comparisons using the Benjamini-Hochberg false discovery rate (FDR) procedure, applied separately per contrast family (within-treatment HAD, within-treatment TAD, and between-treatment DiD). Significance was declared at FDR < 0.05. Model convergence and singularity were logged for every feature to identify unreliable fits.

**Multi-omic integration: DIABLO**

Supervised multi-omic integration was performed using Data Integration Analysis for Biomarker discovery using Latent cOmponents, implemented in the mixOmics R. DIABLO extends sparse partial least squares discriminant analysis (sPLS-DA) to multiple data blocks, identifying correlated features across omic layers that jointly discriminate between conditions.

**Input data preparation**

For each participant and treatment, within-treatment change scores (Δ = Post diet - Baseline) were computed from the CLR-transformed microbiome data and log-transformed metabolomics data. Three predictor blocks were constructed: KO gene families, plasma metabolites, and urine metabolites. Only participants with complete data across all three blocks for both diets were retained. All blocks were z-score standardised (mean-centred, unit-variance scaled) to ensure comparability across platforms with different measurement scales. The outcome variable (Y) was the dietary treatment label (HAD/TAD) for each participant-treatment observation. Zero-variance features were removed prior to modelling.

**Design matrix**

The DIABLO design matrix encodes expected correlations between blocks. Connections between KO and metabolite blocks (plasma, urine) were set to 0.8, reflecting the biological prior that microbial enzyme-coding genes catalyse metabolic reactions detected as plasma and urine metabolites. Inter-metabolite block connections were set to 0.1.

**Feature selection and tuning**

The number of features to retain per block per component (keepX) was tuned using subject-blocked 5-fold cross-validation with 10 repeats. Subject blocking ensured that both diet observations from the same participant remained in the same fold, preventing data leakage from within-subject correlation. KeepX values were tested over a grid of 5, 10, 15, and 20 features for KO, and 10, 20, 30, and 40 for plasma and urine metabolites. The combination minimising the balanced error rate (BER) on held-out folds was selected. Two latent components were extracted.

**Classification performance and permutation testing**

Classification accuracy was assessed in two stages. First, the optimal keepX was selected via subject-blocked 5-fold cross-validation (10 repeats) with max-distance prediction, minimising the balanced error rate (BER). Second, a separate held-out evaluation was conducted: using the selected keepX, a fresh round of subject-blocked 5-fold cross-validation (10 repeats, with different fold assignments from tuning) was performed to obtain an unbiased BER estimate. Statistical significance was evaluated by a permutation test (200 permutations): at each iteration, diet labels were randomly shuffled and cross-validated BER was computed. The permutation p-value was calculated as the proportion of null BER values equal to or lower than the observed held-out BER, with a pseudo-count correction. Given the limited sample size (n = 66 observations), nested cross-validation was not implemented to preserve statistical power.

**Feature extraction and visualisation**

Feature loadings (non-zero sparse weights) were extracted for each block and component, identifying the multi-omic signature discriminating between diets. Metabolite features were annotated with chemical names and pathway classifications. Circos plots were generated to visualise inter-block Pearson correlations (r > 0.7). Block agreement was assessed via pairwise scatter plots of component scores with Pearson correlations and 95% confidence ellipses per diet group.

**Multi-omic regression: block.spls**

To identify multi-omic features predicting cardiometabolic improvement, a block sparse partial least squares regression (block.spls) model was fitted using the mixOmics package. Unlike DIABLO, which performs classification, block.spls performs multi-response regression, asking which omic features covary with continuous clinical outcomes. To assess whether composite multi-omic scores generalise beyond the training data, leave-one-out cross-validation was performed: for each of the 32 subjects, a block.spls model was trained on the remaining 31, and the held-out subject’s component scores were predicted from their omic data alone. These out-of-sample scores were then correlated with clinical outcomes using Spearman rank correlations. To account for features with zero variance in individual LOO folds, near-zero-variance features were filtered per fold, with keepX adjusted accordingly.

**Input data preparation**

Within-subject difference-of-differences were computed: for each participant, the TAD change score (ΔTAD = Post diet - Baseline) was subtracted from the HAD change score (ΔHAD), yielding a single row per subject representing the differential effect of the dietary intervention. This design produces truly independent observations (n = 32 subjects), eliminating within-subject correlation. Three predictor (X) blocks were constructed: KO, plasma metabolites, and urine metabolites. The response (Y) matrix comprised nine cardiometabolic outcomes (SBP, DBP, total cholesterol, LDL, HDL, non-HDL, total:HDL ratio, triglycerides, glucose), all expressed as within-subject difference-of-differences. All blocks were z-score standardised.

**Model specification**

The block.spls model was fitted with two components in regression mode, with sparse feature selection retaining 15 features per block per component (adjusted downward if fewer features were available). The design matrix was identical to that used for DIABLO.

**Univariate association screening**

To validate multivariate selections, Spearman rank correlations were computed between each block.spls-selected omic feature and each clinical outcome. FDR correction (Benjamini-Hochberg) was applied across all feature-outcome pairs. Associations at FDR < 0.2 were retained for the feature-outcome heatmap; those at FDR < 0.05 were considered statistically significant.

**Visualisation and reproducibility**

A colourblind-safe palette was used throughout. Paired spaghetti plots, boxplots, PCoA ordinations, change-vector plots, forest plots, volcano plots, circos plots, lollipop loading charts, and correlation heatmaps were generated using ggplot2. Multi-panel figures were assembled using the patchwork R package.

**Multiple testing correction strategy**

A tiered approach to multiple testing correction was adopted. For per-feature differential analyses involving hundreds to thousands of simultaneous tests (species, pathways, metabolites), the Benjamini-Hochberg FDR procedure was applied at a threshold of 0.05, with FDR correction applied separately per contrast family (within-HAD, within-TAD, between-treatment DiD), reflecting the distinct scientific questions addressed by each comparison. This approach controls the false discovery rate within each question at the cost of a modestly elevated study-wide error rate. For alpha diversity, where only four pre-specified, mathematically correlated metrics were tested, raw p-values were reported without adjustment. For block.spls univariate validation, FDR was applied across all feature-outcome pairs with a discovery threshold of 0.2 for visualisation and 0.05 for significance.

**Software and packages**

All analyses were conducted in R (version 4.4.2; R Core Team, 2024). The following key packages were used:

| **Package** | **Purpose** |
| --- | --- |
| tidyverse (v2.0.0) | Data manipulation and visualisation |
| lme4 (v1.1.36) | Linear mixed-effects models |
| lmerTest (v3.1.3) | Satterthwaite degrees of freedom, Type III tests |
| emmeans (v2.0.0) | Estimated marginal means and contrasts |
| vegan (v2.6.10) | PERMANOVA, PERMDISP, ordination |
| mixOmics (v6.32.0) | multi-omics integration: DIABLO, block.spls |
| ggrepel (v0.9.6) | Non-overlapping text labels |
| patchwork (v1.3.2) | Multi-panel figure assembly |
| writexl (v1.5.4) | Excel output |
| readxl (v1.4.4) | Excel input |

**Table S1. Participant characteristics at the first study visit among individuals with complete paired microbiome and metabolomic data (n = 32).**

| **Characteristic** | **Value** |
| --- | --- |
| **Demographic variables (mean, SD or n (%))** |  |
| Sex, female *n* (%) | 17 (53.1%) |
| Age, years | 38.3 ± 18.2 |
| Born in Australia, *n* (%) | 22 (68.8%) |
| **Anthropometric variables (mean, SD)** |  |
| Weight, kg | 76.8 ± 16 |
| Body Mass Index (BMI), kg/m² | 26.5 ± 5.5 |
| **Dietary variables (median, IQR)*** |  |
| Core foods (%kJ) | 70 (62-80.1) |
| Non-core foods (%kJ) | 30 (19.9-38) |
| Vegetables (servings/day) | 3.1 (2.1-4.5) |
| Grains (servings/day) | 2.8 (2.1-3.8) |
| Fruits (servings/day) | 1.3 (0.9-2.1) |
| Seafood (servings/day) | 0.3 (0.1-0.6) |
| Red meat (servings/day) | 0.6 (0.3-0.8) |
| Dietary fibre (g/day) | 29.1 (21.8-33.9) |
| Energy (kJ/day) | 9105 (6703.8-10944.1) |
| Protein (g/day) | 107.6 (67.5-119.2) |
| Added sugars (g/day) | 32.3 (15.7-45.8) |
| Saturated fat (g/day) | 27.5 (20.7-33.2) |
| Sodium (mg/day) | 1840.1 (1534.2-2341.9) |

*Dietary variables derived from assessment using the Australian Eating Survey®. Core foods are defined as per the Australian Dietary Guidelines as those belonging to the five shared food groups (grains, vegetables, fruit, dairy, and lean meats/alternatives), while non-core foods were defined as energy-dense, nutrient-poor “discretionary” choices high in saturated fat, added sugars, and salt. %kJ = percentage energy.

**Table S2.** **DIABLO classification performance and permutation testing.** The model achieved strong discrimination between HAD and TAD dietary responses (held-out balanced error rate [BER] = 0.08, 91.7% accuracy) across both components, using subject-blocked five-fold cross-validation with 10 repeats conducted separately from the tuning procedure. Permutation testing (195 permutations) confirmed that the observed accuracy exceeded chance expectation (permutation p = 0.005; null BER = 0.494).

| **Comp** | **BER (tuning)** | **BER (held-out)** | **BER held-out (sd)** | **Accuracy** | **Method** | **Folds** | **Repeats** | **Perm**  **p-value** | **Perm (n)** | **Perm BER (mean)** |
| --- | --- | --- | --- | --- | --- | --- | --- | --- | --- | --- |
| 1 | 0 | 0.0830 | 0.0830 | 91.7% | Subject-blocked CV (held-out evaluation) | 5 | 10 | 0.005 | 195 | 0.494 |
| 2 | 0 | 0.0830 | 0.0830 | 91.7% | Subject-blocked CV (held-out evaluation) | 5 | 10 | 0.005 | 195 | 0.494 |

**
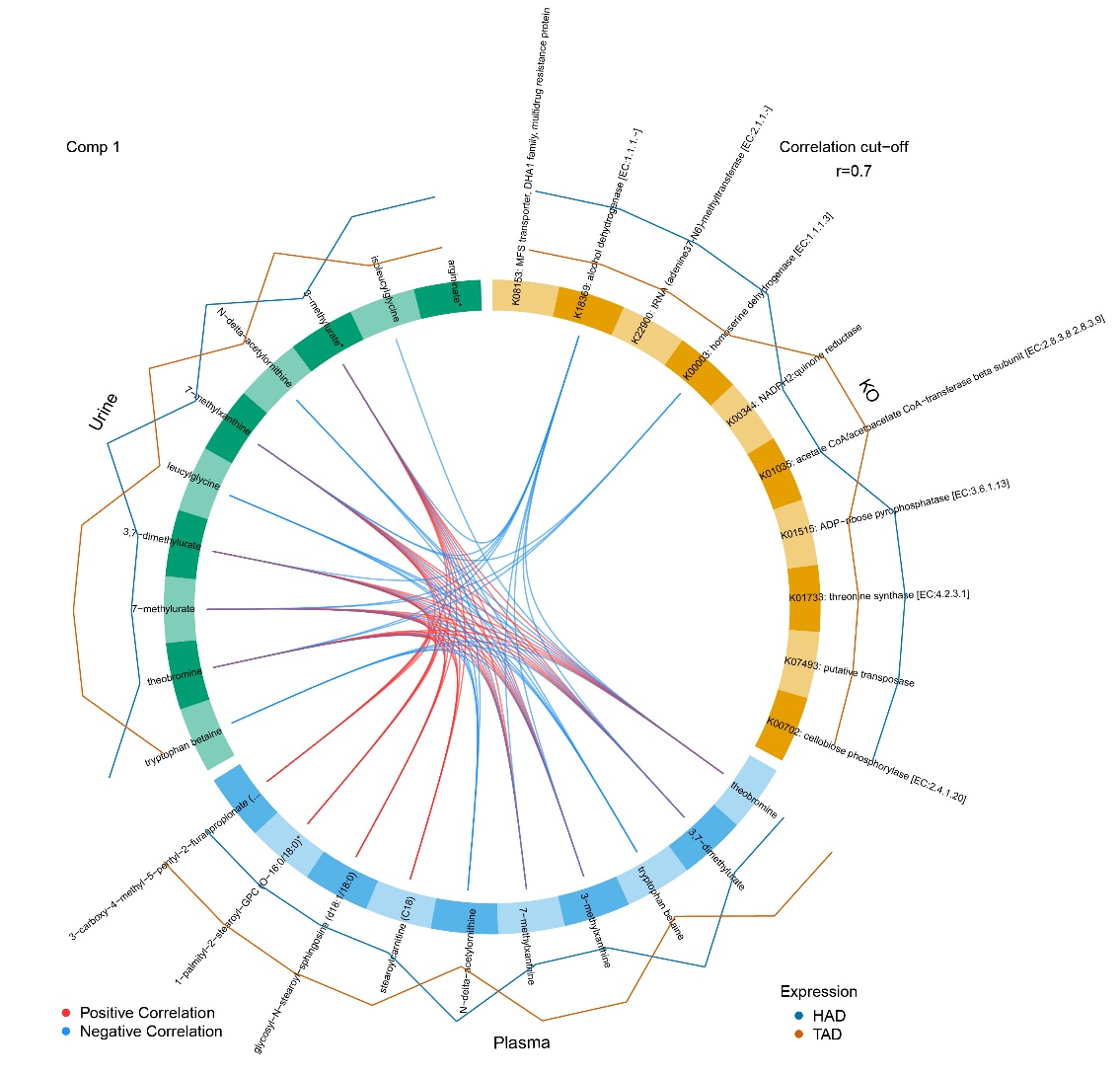
**

**Figure S1.** **Inter-block feature correlations from DIABLO multi-omic integration.** Circos plot displaying pairwise Pearson correlations (r > 0.7) between DIABLO-selected features across three omic blocks: microbial-encoded genes (KO), plasma metabolites, and urine metabolites. Outer ring segments represent each block, with the outer track indicating the mean change-from-baseline for each feature by diet (HAD, blue; TAD, orange): bars extending outward indicate features that increased more from baseline under that diet. Inner links indicate strong positive (red) or negative (blue) correlations between features across blocks. The model was fitted on within-treatment change scores (Δ = post-diet - baseline) from n = 64 observations (32 subjects × 2 diets), with 10 features retained per block per component.


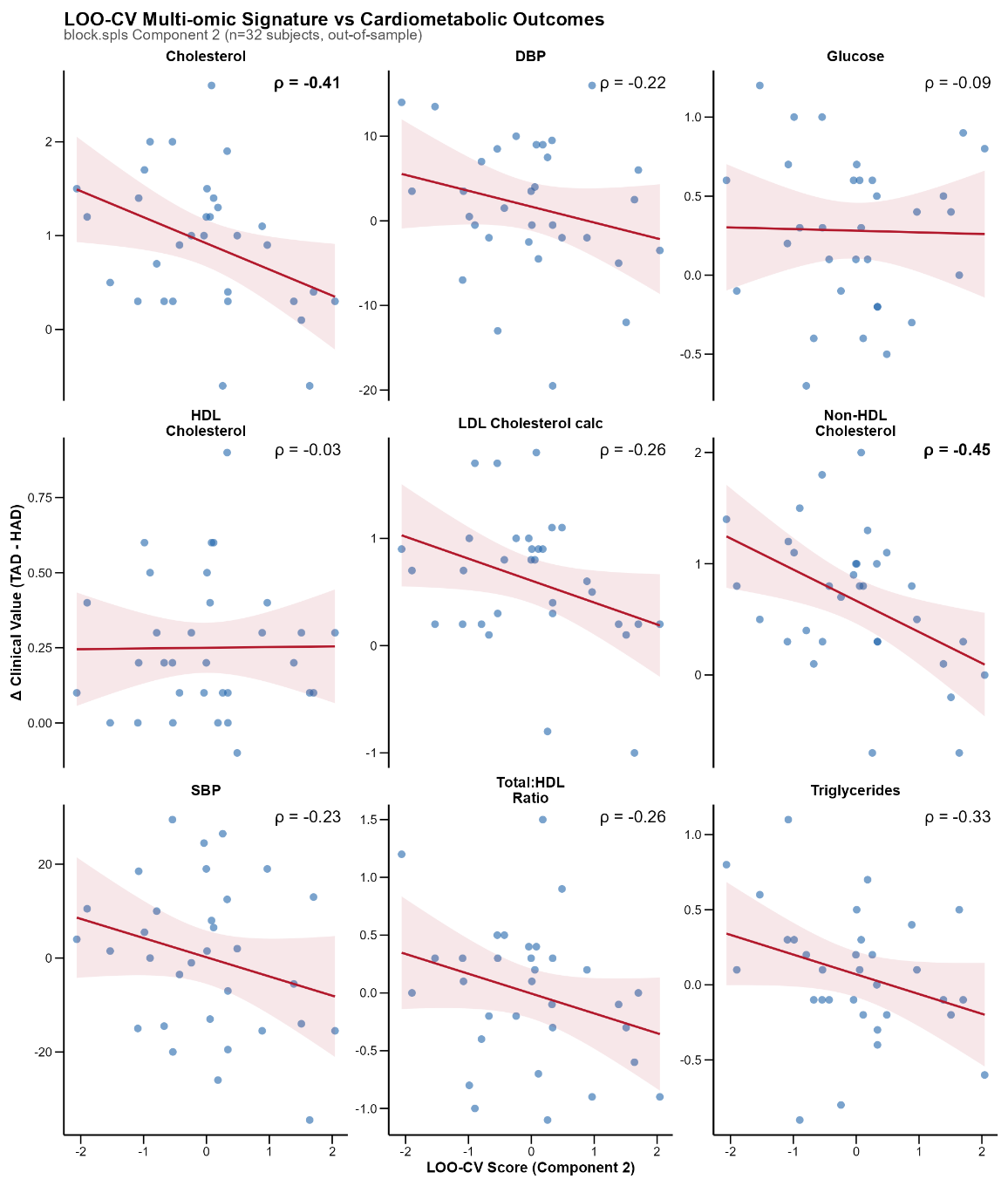


**Figure S2. Leave-one-out cross-validated multi-omic scores versus cardiometabolic outcomes (Component 2).** Scatterplots show the relationship between each participant’s out-of-sample Component 2 composite multi-omic score, predicted via leave-one-out cross-validation of the block.spls model, and their between-diet difference (Δ TAD - Δ HAD) in each cardiometabolic marker (n = 32 subjects). Red lines represent the linear trend with 95% confidence intervals. Spearman correlations are shown per panel, with significant associations (p < 0.05) in bold. Component 2 significantly predicted out-of-sample variation in total cholesterol (ρ = -0.41, p = 0.02) and non-HDL cholesterol (ρ = -0.44, p = 0.01), with a borderline association with triglycerides (ρ = -0.33, p = 0.06).
